# Effect of Hydroalcoholic Extract of *Cydonia oblonga* Miller (Quince) on Sexual Behaviour of Wistar Rats

**DOI:** 10.1155/2014/282698

**Published:** 2014-02-04

**Authors:** Muhammad Aslam, Ali Akbar Sial

**Affiliations:** ^1^Department of Basic Medical Sciences, Faculty of Pharmacy, Ziauddin University, Karachi 75600, Pakistan; ^2^Department of Pharmaceutics, Faculty of Pharmacy, Ziauddin University, Karachi 75600, Pakistan

## Abstract

*Cydonia oblonga* Miller (quince) is regarded as a potent libido invigorator in Tib-e-Nabvi and Unani System of Medicine. This study was carried out to evaluate the aphrodisiac activity of the hydroalcoholic extract of the fruits of *Cydonia oblonga* Miller (quince) in Wistar rats. The extract was administered orally by gavage in the dose of 500 mg/kg and 800 mg/kg body weight per day as a single dose for 28 days. The observed parameters were mounting frequency, assessment of mating performance, and orientation activities towards females, towards the environment, and towards self. The results showed that after administration of the extract mounting frequency and the mating performance of the rats increased highly significantly (P < 0.01). The extract also influenced the behaviour of treated animals in comparison to nontreated rats in a remarkable manner, making them more attracted to females. These effects were observed in sexually active male Wistar rats.

## 1. Introduction

Male infertility is mostly caused by abnormalities in the male reproductive system. These abnormalities include impotence, erectile dysfunction, premature ejaculation, and decreased sexual desire. Sexual desire is an inescapable function of life. The principal role of sex and sexuality is the “continuation of progeny” and the survival of living organisms [1]. An aphrodisiac substance is a drug which enhances sexual desire. Etymologically, the term aphrodisiac had been derived from *Aphrodite*, the Greek goddess of sexuality and love [2]. There are a number of allopathic drugs used to enhance sexual desire in males and females, but these drugs have various adverse effects [3]. Today, most commonly used aphrodisiac drugs are phosphodiesterase type 5 inhibitors such as sildenafil (Viagra) and tadalafil. The adverse reactions produced by sildenafil and tadalafil include transient headache, dyspepsia, flushing, diarrhoea, dizziness, pulse irregularities, visual disturbance, and priapism.

Therefore, it is needed to explore the newer aphrodisiac drugs with a better safety profile. Traditional herbal drugs have proven to be a better choice when compared to modern synthetic drugs. These drugs have a few or no side effects and are claimed to be safer ones [4]. However, certain studies have shown that herbal drugs have the potential to interact with other drugs and food [5–10]. *Cydonia oblonga *Miller (quince) family Rosaceae also known as bahi (Urdu) and safarjal (Arabic) is official in Tib-e-Nabvi and is mentioned in the Holy Quran [11]. Traditionally, *Cydonia oblonga* had been used as an antidiarrhoeal, gastric tonic, ulcer-healing, anti-inflammatory, antiemetic and astringent agent. The fruit is suitable for uterine and hemorrhoid bleeding [12]. A number of pharmacological studies have also revealed antimicrobial activity [13], antiradical activity [14], antioxidant activity [15], the inhibitory effect on IgE immune reactions [16], antiulcerative activity [17], antiproliferative activity [18], antihemolytic activity [15], antiallergic activity [19], lipid-lowering activity [20], antidiabetic activity [21], and healing effects of *Cydonia oblonga* [22]. This study was carried out to evaluate the aphrodisiac activity of the hydroalcoholic extract of *Cydonia oblonga* Miller. To the best of our knowledge, this is the first study on the aphrodisiac effects of *Cydonia oblonga* Miller.

## 2. Materials and Methods

### 2.1. The Collection of Plant Material

Fresh fruits of *Cydonia oblonga* Miller, *at mature stage*, were purchased from local markets in Malir, Karachi 24°51N 67°02E, in November 2012. The sample was authenticated by an herbalist of the Department of Herbal Extracts, Avicenna Foundation, Pakistan. Voucher specimen (RP/PHARM/1103) was deposited in the institute for future reference.

### 2.2. Preparation of Hydroalcoholic Extract

The fruits of *Cydonia oblonga* along with their peels were cut into slices and shade-exsiccated at room temperature. Fine powder was made from 200 grams of the slices and soaked in sufficient volume of ethanol/water (70/30) for 1 hour. Further extraction was carried out using a percolator for 72 hours to complete the extracting process [23]. The extract was then subjected to filtration and made solvent-free using a rotary evaporator. To obtain a semisolid concentrated extract, the fluid extract obtained in the preceding step was further freeze-dried until a dry powder was produced [24].

### 2.3. Drugs and Chemicals

Sildenafil citrate (PZ0003 SIGMA), estradiol benzoate (46552 FLUKA), and progesterone (P0130 SIGMA) were purchased from Sigma-Aldrich.

### 2.4. The Selection of Animals

Male albino rats (Wistar strain) weighing between 150 and 200 g were used in this research. The specifications given in Helsinki Resolution 1964 were followed during animal handling. This research was approved by our institutional ethical committee vide Resol. number 12/PHA/89.

### 2.5. Determination of Acute Oral Toxicity

The extract in the dose range of 500–3000 mg/kg was given through oral route to different groups of rats. The control group received distilled water. Animals were kept in fasting condition before the administration of the doses. Following the period of fasting, the animals were weighed and properly marked and the extract was administered. After administration of the extract, food was withheld for two hours. Litchfield and Wilcoxon method was used to determine the acute toxicity. Acute toxicity study of the extract showed that the extract in the present investigation was nontoxic up to 3000 mg/kg body weight [25].

### 2.6. Dosing

The dose of the extract was calculated according to the body weight of the animals. The dosing of the drug was done daily in normal doses according to the body weight of the animals.

### 2.7. Methodology

Healthy adult albino rats (Wistar strain) weighing 150–200 g were procured from animal house of University of Karachi, Pakistan. Before administration of the drug, the animals were kept in the laboratory for one week for the conditioning period. They were kept individually in polypropylene cages under controlled conditions at room temperature (25–30°C) with 12/12 hours light-dark cycle. The rats were given a standard rat diet and water *ad libitum*.

#### 2.7.1. Preparation of Male Rats

Male rats were given training, for sexual behaviour, twice a day for a period of 10 days. In case a rat showed a lack of sexual interest during the test period, it was considered as an inactive male and was replaced by another sexually active rat [26].

#### 2.7.2. Preparation of Female Rats

The female rats were artificially brought into oestrus (heat) by the sequential administration of estradiol benzoate (10 *μ*g/100 g body weight) and progesterone (0.5 mg/100 g body weight) through subcutaneous injections, 48 hours and 4 hours, respectively, before mating. After the confirmation of receptivity of the female rats, the experiment was started [26].

#### 2.7.3. Experimental Details

The sexually active male rats were divided into four groups of six animals as follows.

 Group I: normal control, given vehicle (distilled water, 2 mL/kg) orally for 28 days;
 Group II: treated group, given hydroalcoholic extract at the dose of 500 mg/kg (orally, 2 mL/kg) for 28 days;
 Group III: treated group, given hydroalcoholic extract at the dose of 800 mg/kg (orally, 2 mL/kg) for 28 days;
 Group IV: standard drug group, given sildenafil citrate at the dose of 5 mg/kg (orally, 2 mL/kg) for 28 days.

To determine the aphrodisiac activity of the extract, several parameters were observed. These include measuring and observing the mounting frequency, assessment of mating performance, and orientation activities towards females, towards the environment, and towards self.

#### 2.7.4. Mounting Behaviour Test

The mount is operationally defined as the male assuming the copulatory position, but failing to achieve intromission. To quantify mounting frequency, mounting behaviour test was used. Non-oestrus female rats were paired with males treated with the drug (500 mg/kg and 800 mg/kg; p.o.). Animals were observed for 3 hours and their behaviours were scored on the 14th and the 28th day of dosing. Males were placed individually in glass cages. After 15 minutes of acclimatization, a non-oestrus female was introduced into each cage. The number of mounts were noted during a 15-minute observation period at the start of 1st hour. Then the female was withdrawn from each cage for a period of 105 minutes. Again, the female was introduced and the number of mounts were observed for 15 minutes as before at 3rd hour. All the experiments were carried out between 9:00 a.m. to 12:00 p.m. during daytime at room temperature 26-27°C [27, 28].

#### 2.7.5. Assessment of Mating Performance

Males were placed individually in glass cages. After 15 minutes of acclimatization, five oestrus females were admitted into each cage and they cohabited overnight. Microscopic examination of the vaginal smear of each female mouse was done to detect the presence of any sperms. The number of sperm positive females was recorded in each group [28].

#### 2.7.6. Assessment of the Orientation Activities of Male Rats

The orientation activities of male rats towards females, towards the environment, and towards self were evaluated. After thirty minutes of the administration of the extract, the rats were observed for the next one hour and the number of lickings, anogenital sniffings, climbings, and genital groomings was counted for one hour [2].

#### 2.7.7. Statistical Analysis

All values are mean ± standard deviation (SD). All values were compared with control and standard drug. The significance of difference in the mean was determined by Student's *t*-test. Values of *P* < 0.05 were considered as significant and *P* < 0.01 as highly significant. The data were analysed by using SPSS program Version 20.

## 3. Results

### 3.1. Effect of *Cydonia oblonga* on the Mounting Behaviour of Male Rats

Male rats treated with *Cydonia oblonga* (500 mg/kg and 800 mg/kg) showed a highly significant increase (*P* < 0.01) in the mounting behaviour after 1 hour and 3 hours of drug administration when compared to normal control group. The effect was observed on the 14th and the 28th day of the drug treatment. The increase in the number of mounts produced by *Cydonia oblonga* extract was comparable to the standard drug (Tables 1 and 2).

### 3.2. Effect of *Cydonia oblonga* on the Mating Performance of Male Rats

**Figure 1:**
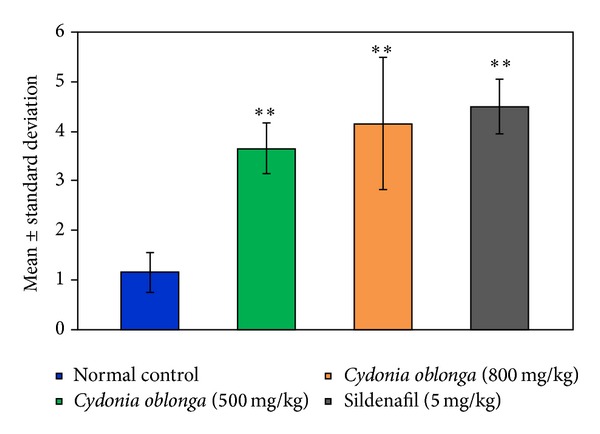
Effect of *Cydonia oblonga* on the mating performance of male rats on the 14th day of the study.

After administration of the extract of *Cydonia oblonga* (500 mg/kg; p.o.), *Cydonia oblonga* (800 mg/kg; p.o.), and sildenafil citrate (5 mg/kg; p.o.) there was observed an increase in the mating performance of the rats. On the 14th day of the study, out of six animals in Group I (normal control), one male mated with (inseminated) two females and the remaining five males mated with one female each during the overnight experimental period. However, in Group II (*Cydonia oblonga* 500 mg/kg; p.o.), two males mated with three females and the remaining four rats mated with four females each. In Group III, (*Cydonia oblonga* 800 mg/kg; p.o.), one male mated with three females, one male mated with two females, and the remaining four rats mated with five females each. In Group IV (standard drug group), three males mated with five females each and three males mated with four females each. The mean number of females (mean ± SD) mated by one male in the control group was 1.16 ± 0.40 while it was 3.66 ± 0.51 (*P* < 0.001), 4.16 ± 1.33 (*P* < 0.001), and 4.50 ± 0.55 (*P* < 0.001) in the groups treated by *Cydonia oblonga* (500 mg/kg; p.o.),* Cydonia oblonga* (800 mg/kg; p.o), and sildenafil citrate, respectively (Figure 1).

**Figure 2:**
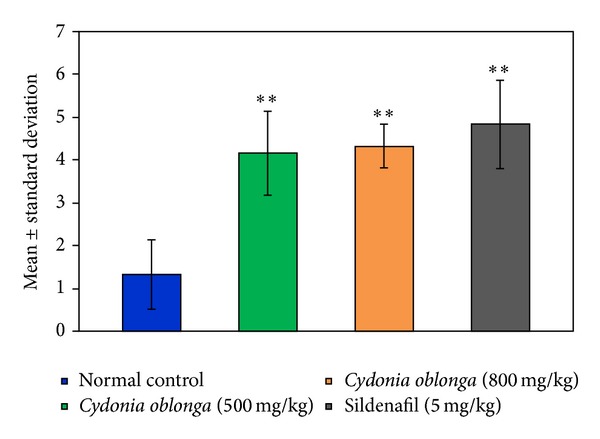
Effect of *Cydonia oblonga* on the mating performance of male rats on the 28th day of the study.

On the 28th day of the study, out of six animals in Group I (normal control), only one male mated with (inseminated) three females and the remaining five males mated with one female each during the overnight experimental period. Whereas, in Group II (*Cydonia oblonga* 500 mg/kg; p.o.), two males mated with three females, one male mated with four females, and the remaining three males mated with five females each. In Group III, (*Cydonia oblonga* 800 mg/kg; p.o.), four males mated with five females each; two males mated with three females each. In Group IV (standard drug group), five males mated with five females each and one male mated with four females. The mean number of females (mean ± SD) mated by one male in the control group was 1.33 ± 0.81 while it was 4.16 ± 0.98 (*P* < 0.001), 4.66 ± 0.51 (*P* < 0.001), and 4.33 ± 1.03 (*P* < 0.001) in the groups treated by *Cydonia oblonga* (500 mg/kg; p.o.),* Cydonia oblonga* (800 mg/kg; p.o.), and sildenafil citrate, respectively, (Figure 2).

### 3.3. Effect of *Cydonia oblonga* on the Orientation Activities of Male Rats

The extract also influenced the behaviour of treated animals in a noteworthy manner, making them more attracted to females. The assessment of general parameters such as licking, anogenital sniffing, climbing, and genital grooming also proved the aphrodisiac activity of the plant.

#### 3.3.1. Licking

On the 14th day of the study, the results of licking showed a significant (*P* < 0.05) increase in the animals treated with hydroalcoholic extract at 800 mg/kg body weight. Whereas there was only an insignificant increase at 500 mg/kg body weight. However, on the 28th day a significant increase (*P* < 0.05) in the number of lickings was observed in the animals treated with hydroalcoholic extract 500 mg/kg body weight and a highly significant increase (*P* < 0.01) in the number of lickings was observed in the animals treated with hydroalcoholic extract at 800 mg/kg body weight (Tables 3 and 4).

#### 3.3.2. Anogenital Sniffing

On the 14th and the 28th day of the study, the results of anogenital sniffing showed a highly significant (*P* < 0.01) increase in the animals treated with hydroalcoholic extract at 800 mg/kg body weight. Whereas a significant (*P* < 0.05) increase in the number of anogenital sniffings was observed in the animals treated with hydroalcoholic extract at 500 mg/kg body weight (Tables 3 and 4).

#### 3.3.3. Climbing

On the 14th and the 28th day of the study, the results of climbing showed a highly significant (*P* < 0.01) increase in the animals treated with hydroalcoholic extract at 800 mg/kg body weight. Whereas a significant (*P* < 0.05) increase in the number of climbings was observed in the animals treated with hydroalcoholic extract at 500 mg/kg body weight (Tables 3 and 4).

#### 3.3.4. Genital Grooming

On the 14th and the 28th day of the study, the results of genital grooming exhibited a highly significant (*P* < 0.01) increase in the animals treated with hydroalcoholic extract at 800 mg/kg body weight. Whereas a significant (*P* < 0.05) increase in the number of genital groomings was observed in the animals treated with hydroalcoholic extract at 500 mg/kg body weight (Tables 3 and 4).

## 4. Discussion

*Cydonia oblonga *Miller (quince) is regarded as a potent libido invigorator in Tib-e-Nabvi and Unani System of Medicine. The results of the current study demonstrated that after administration of the hydroalcoholic extract the sexual activity of male Wistar rats was increased highly significantly. The aphrodisiac potential of *Cydonia oblonga *may be due to its secondary metabolites such as flavonoids, glycosides, tannins, and phenolic compounds present in the extract. It has been observed that drug-induced alterations in the levels of neurotransmitters or their actions at the cellular level could also alter sexual behaviour [29]. Limbic system is the area of the human brain that has the main regulatory role in sexual behaviour. The literature shows a very close relationship between dopamine and 5-hydroxytryptamine and sexual behaviour [30]. The association of human sexual behaviour with dopamine is substantiated by reports of sexual behaviour induced by L-dopa in Parkinsonian patients. Stimulant drugs and antidepressant drugs have been well known to affect libido, erection, ejaculation, and orgasm. Dopamine is one of the most extensively studied central neurotransmitters involved in the control of sexual behaviour. As a matter of fact, while the nigrostriatal system is important for the control of the sensory-motor coordination needed for copulation, the mesolimbic-mesocortical system performs a key role in the preparatory phase of the behaviour, principally in sexual arousal, motivation, and possibly reward. The dopaminergic receptors, which are involved in the control of male sexual behaviour, belong to the D2 receptor subtype. Most studies have shown that drugs, which increase dopaminergic transmission, improve male sexual behaviour, and those drugs, which decrease dopaminergic transmission, worsen male sexual behaviour [31]. *Cydonia oblonga* is a good and cheap natural source of potent antioxidants such as phenolic acids and flavonoids [32, 33]. The fruits of *Cydonia oblonga* contain phenolic compounds, including chlorogenic acid, which is the principal phenolic compound of the fruits and has potent antioxidant [34] as well as anti-inflammatory [29] effects which can halt edema, inflammation, neutrophil migration, and TNF-*α* expression [35, 36]. Rutin, quercetin, and kaempferol are the well-known quince flavonoids with very potent antioxidant and immunomodulatory effects [37, 38]. Flavonoids react with free radicals and form more stable radicals with lower toxicity. Flavonoids also cause chelation of Fe^2+^ that results in the inhibition of the effects of free radicals [38]. These antioxidants act as cell saviours through their abilities as reducing agents, hydrogen donators, free radical scavengers, and singlet oxygen quenchers [13]. Therefore, it may be that the antioxidative potential of *Cydonia oblonga* might have protected the dopaminergic neurons, including serotenergic and adrenergic neurons against oxidative stress and their number has increased; correspondingly sexual behaviour has also been increased. The free radical scavenging potential of quince is associated with its antihemolytic activities [15] so it may be that the positive effects of the drug on blood are involved in its aphrodisiac activity.

## 5. Conclusion

Based on the results of our study, we conclude that the oral use of hydroalcoholic extract of *Cydonia oblonga *has sexual behaviour-enhancing effect in male Wistar rats. However, the mechanism of the sexual behaviour enhancing-effect of this miracle herb is yet to be elucidated.

## Figures and Tables

**Table 1:** Effect of *Cydonia oblonga* on the mounting behaviour of male rats on the 14th day of the study.

Group	Number of mounts per 15 minutes	
	1st hour	3rd hour
Normal control	2.2 ± 0.61	1.3 ± 0.40
Treated group (500 mg/kg)	5.4 ± 0.20^a^	6.9 ± 0.51^a^
Treated group (800 mg/kg)	9.4 ± 0.27^a^	10.3 ± 0.92^a^
Standard group (5 mg/kg)	12.7 ± 0.73^a^	13.9 ± 0.22^a^

^a^ *P* < 0.01: significant difference when compared with the control group.

Values are mean ± standard deviation.

**Table 2:** Effect of *Cydonia oblonga* on the mounting behaviour of male rats on the 28th day of the study.

Group	Number of mounts per 15 minutes	
	1st hour	3rd hour
Normal control	1.7 ± 0.54	1.9 ± 0.76
Treated group (500 mg/kg)	7.3 ± 0.49^a^	7.9 ± 0.41^a^
Treated group (800 mg/kg)	12.2 ± 0.46^a^	11.9 ± 0.72^a^
Standard group (5 mg/kg)	13.5 ± 0.35^a^	15.4 ± 0.63^a^

^a^ *P* < 0.01: highly significant difference when compared with the control group.

Values are mean ± standard deviation.

**Table 3:** Effect of *Cydonia oblonga* extract on orientation activities of male rats towards females, towards environment, and towards self on the 14th day of the study.

Groups	Towards female (1 hour)		Towards environment (1 hour)	Towards self (1 hour)
	Licking	Anogenital sniffing	Climbing	Genital grooming
Normal control	3.12 ± 0.14	2.92 ± 0.59	9.85 ± 0.78	6.33 ± 0.29
Treated group (500 mg/kg)	3.66 ± 0.78^IS^	3.51 ± 0.35^a^	10.76 ± 0.49^a^	6.74 ± 0.31^a^
Treated group (800 mg/kg)	4.13 ± 0.23^a^	3.71 ± 0.15^b^	11.92 ± 0.33^b^	8.18 ± 0.47^b^
Standard group	5.82 ± 0.45^b^	6.31 ± 0.74^b^	14.56 ± 0.32^b^	9.66 ± 0.27^b^

Values are mean ± standard deviation.

^a^ *P* < 0.05: significant difference when compared with the control group.

^b^ *P* < 0.01: highly significant difference when compared with the control group.

IS: insignificant difference when compared with the control group.

**Table 4:** Effect of *Cydonia oblonga* extract on orientation activities of male rats towards females, towards environment, and towards self on the 28th day of the study.

Groups	Towards female (1 hour)		Towards environment (1 hour)	Towards self (1 hour)
	Licking	Anogenital sniffing	Climbing	Genital grooming
Normal control	3.23 ± 0.21	2.61 ± 0.13	8.69 ± 0.43	5.85 ± 0.71
Treated group (500 mg/kg)	3.98 ± 0.78^a^	3.20 ± 0.41^a^	9.41 ± 0.53^a^	7.62 ± 0.58^b^
Treated group (800 mg/kg)	4.73 ± 0.17^b^	3.98 ± 0.54^b^	12.29 ± 0.18^b^	8.67 ± 0.53^b^
Standard group	7.11 ± 0.45^b^	8.73 ± 0.74^b^	15.40 ± 0.93^b^	10.34 ± 0.69^b^

Values are mean ± standard deviation.

^a^ *P* < 0.05: significant difference when compared with the control group.

^b^ *P* < 0.01: highly significant difference when compared with the control group.

IS: insignificant difference when compared with the control group.
